# Pharmacological but not physiological GDF15 suppresses feeding and the motivation to exercise

**DOI:** 10.1038/s41467-021-21309-x

**Published:** 2021-02-15

**Authors:** Anders B. Klein, Trine S. Nicolaisen, Niels Ørtenblad, Kasper D. Gejl, Rasmus Jensen, Andreas M. Fritzen, Emil L. Larsen, Kristian Karstoft, Henrik E. Poulsen, Thomas Morville, Ronni E. Sahl, Jørn W. Helge, Jens Lund, Sarah Falk, Mark Lyngbæk, Helga Ellingsgaard, Bente K. Pedersen, Wei Lu, Brian Finan, Sebastian B. Jørgensen, Randy J. Seeley, Maximilian Kleinert, Bente Kiens, Erik A. Richter, Christoffer Clemmensen

**Affiliations:** 1 Novo Nordisk Foundation Center for Basic Metabolic Research, Faculty of Health and Medical Sciences, University of Copenhagen, Copenhagen, Denmark; 2 Section of Molecular Physiology, Department of Nutrition, Exercise and Sports, Faculty of Science, University of Copenhagen, Copenhagen, Denmark; 3 Department of Sports Science and Clinical Biomechanics, University of Southern Denmark, Odense, Denmark; 4 Department of Clinical Pharmacology, Bispebjerg-Frederiksberg Hospital, University of Copenhagen, Copenhagen, Denmark; 5 Centre of Inflammation and Metabolism, Centre for Physical Activity Research, Rigshospitalet, University of Copenhagen, Copenhagen, Denmark; 6 Xlab, Center for Healthy Aging, Department of Biomedical Sciences, Faculty of Health and Medical Sciences, University of Copenhagen, Copenhagen, Denmark; 7 Novo Nordisk Research Center Indianapolis, Indianapolis, IN USA; 8 Global Obesity and LD Research, Novo Nordisk A/S, Maaloev, Denmark; 9 Department of Surgery, University of Michigan, Ann Arbor, MI USA; 10 Institute for Diabetes and Obesity, Helmholtz Diabetes Center at Helmholtz Zentrum München, German Research Center for Environmental Health, Neuherberg, Germany

**Keywords:** Homeostasis, Molecular medicine

## Abstract

Growing evidence supports that pharmacological application of growth differentiation factor 15 (GDF15) suppresses appetite but also promotes sickness-like behaviors in rodents via GDNF family receptor α-like (GFRAL)-dependent mechanisms. Conversely, the endogenous regulation of GDF15 and its physiological effects on energy homeostasis and behavior remain elusive. Here we show, in four independent human studies that prolonged endurance exercise increases circulating GDF15 to levels otherwise only observed in pathophysiological conditions. This exercise-induced increase can be recapitulated in mice and is accompanied by increased *Gdf15* expression in the liver, skeletal muscle, and heart muscle. However, whereas pharmacological GDF15 inhibits appetite and suppresses voluntary running activity via GFRAL, the physiological induction of GDF15 by exercise does not. In summary, exercise-induced circulating GDF15 correlates with the duration of endurance exercise. Yet, higher GDF15 levels after exercise are not sufficient to evoke canonical pharmacological GDF15 effects on appetite or responsible for diminishing exercise motivation.

## Abstract (web summary)

The physiological role of GDF15 remains poorly defined. Here, the authors show that circulating GDF15 increases in response to prolonged exercise, but that this exercise-induced GDF15, unlike pharmacological GDF15, does not affect post-exercise food intake or exercise motivation.

## Introduction

GDF15 was originally discovered as a macrophage inhibitory cytokine (MIC-1)^1^ but in recent years it has attracted considerable attention due to its anti-obesity potential^2^. Administration of recombinant GDF15 to rodents and monkeys promotes substantial anorexia and weight loss^3–7^. However, GDF15 administration also promotes aversive behavior and nausea in rodents^8,9^. Whereas the pharmacological effects of GDF15 have undergone considerable scientific scrutiny, the normophysiological role of endogenous GDF15 on energy homeostasis and behavior remains unknown. We and others have previously shown that vigorous exercise increases circulating GDF15 in humans^10–13^. Given the role of exogenous GDF15 on malaise-related anorexia, we wondered if endogenously-derived GDF15 promotes a similar behavior. Herein, we pursue the hypothesis that exercise-induced GDF15 is an endocrine feedforward signal that protects the organism from excessive physical and/or metabolic stress by signaling exercise aversion through GFRAL. Further, we investigate the possibility that GDF15 is responsible for the temporary anorexia after intense exercise. Our work show that prolonged endurance exercise leads to a 4-5-fold increase in circulating GDF15 in mice and humans and that pharmacological GDF15 supresses voluntary running in mice. The exercise-induced increase in endogenous GDF15 does not affect post-exercise food intake, nor does it signal exercise aversion.

## Results

### Exercise increases circulating GDF15 in humans

**Fig. 1:**
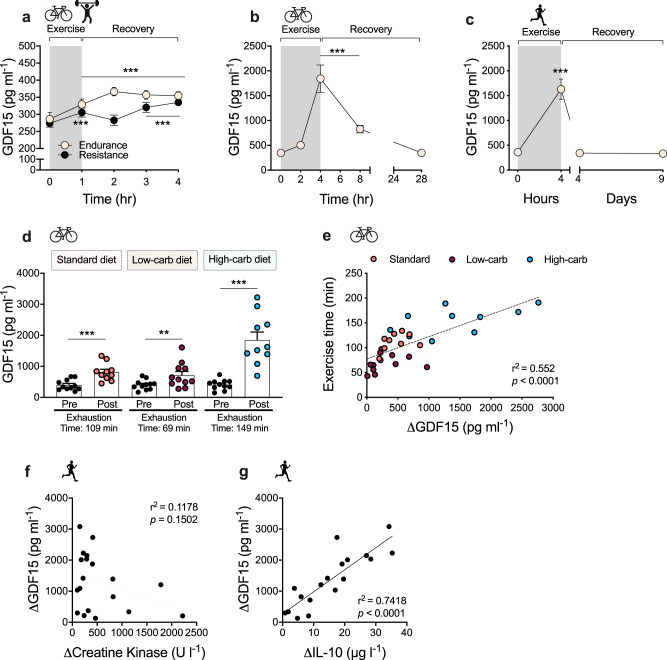
Effect of exercise on circulating GDF15 in humans. **a** Plasma GDF15 values before and after 1 h of high-intensity resistance (*n* = 10) or vigorous endurance exercise (*n* = 10) and at indicated time points during recovery (*p* < 0.001). **b** Plasma GDF15 levels were measured in highly trained elite male triathletes (*n* = 15) bicycling at 73 ± 1% of maximal heart rate (HR_max_) for 4 h, with blood sampling as indicated immediately before, during, and in recovery from exercise (*p* < 0.001), with one missing value at 28 h. **c** Plasma GDF15 levels measured before and after a marathon (42.195 km); completing time was 243 ±  8 min [mean ± SEM] (4 h on the figure), with additional measurements of GDF15 on day 2–5 (*p* < 0.001) and day 9–10 after the marathon (on day 4 and day 9) (*n* = 20). **d** On three separate occasions, under three different dietary regimes as indicated, subjects performed cycling exercise at 75% of maximal oxygen uptake (VO_2max_) until exhaustion. GDF15 plasma levels were measured pre-exercise and at exhaustion on each occasion (*n* = 11; standard diet, *p* < 0.001; low-carb diet, *p* = 0.0064; high-carb diet, *p* < 0.001; one missing value in the high-carb group post-exercise). **e** Relationship between change in GDF15 values (ΔGDF15) and time to exhaustion for the diet-exercise interventions presented in **d**. **f** Relationship between change in GDF15 values (ΔGDF15) (from **c**.) and Δcreatine kinase pre vs. post marathon. **g** Relationship between change in GDF15 values (ΔGDF15) (from **c**) with ΔIL-10 plasma values pre vs. post marathon. Data are presented as mean ±  SEM, *P < 0.05; **P < 0.01; ***P < 0.001 compared to time-point 0 or as indicated. **a** Repeated measures two-way ANOVA with Bonferroni multiple comparisons test. **b** and **c** Repeated measures One-way ANOVA relative to starting time, **d** Two-tailed paired *t* tests for each exercise–diet trial. Source data are provided as a Source Data file.

If GDF15 is a feedforward signal that protects the body from excessive exercise stress, then it should be secreted in response to any type of exercise of sufficient intensity and to a greater extent during prolonged exercise. To investigate this rationale, we first compared the effects of endurance vs. resistance exercise on circulating GDF15 in a randomized crossover study of 1 h of bicycling at 70% of VO_2peak_ or 1 h of high-volume resistance exercise in moderately trained males. Both endurance and resistance exercise increased circulating GDF15 measured immediately after exercise by ~15% (Fig. 1a). During the 3 h post-exercise recovery period, GDF15 plasma levels increased faster following the cycling exercise bout, but levels were similarly increased by ~25% compared to baseline, 3 h after both modes of exercise, suggesting that exercise increases GDF15 independently of exercise mode. We next tested circulating GDF15 concentrations in elite male triathletes cycling for 4 h at 73% of their maximum heart rate and found a remarkable 5.3-fold increase in plasma GDF15 compared to baseline (Fig. 1b). More than 30% of the subjects reached plasma levels higher than 2.5 ng/ml, levels that are usually only seen in pathological conditions^14,15^. Of note, GDF15 levels returned to baseline values within 24 h after exercise. In well-trained subjects, we observed a comparable 4.5-fold increase in circulating GDF15 following the completion of a marathon with an average completion time of 4 h (Fig. 1c). To evaluate the role of exercise duration and substrate availability on exercise-induced GDF15 levels, we analyzed plasma levels of GDF15 in another group of well-trained subjects that were instructed to cycle to exhaustion under three different dietary regimes that subjects adhered to for 72 h prior to the exercise bout: a standard diet, a low-carbohydrate diet, and a high-carbohydrate diet. A carbohydrate-rich diet, which increases muscle glycogen content, delays fatigue substantially during exercise time trials^16^. In this study, the induction of circulating GDF15 was greatest during the high-carbohydrate trial, which also coincided with the longest trial time (149 min, 4.5-fold increase in GDF15) (Fig. 1d). Whereas these data do not allow for making firm conclusions about whether exercise duration per se or certain substrate availability in combination with exhaustive exercise play a role for the exercise-induced GDF15, they do underscore that exercise stress promotes a steep increase in plasma GDF15 once the activity goes beyond 2 h (Fig. 1b, d, e). Conversely, GDF15 was least induced in the low carbohydrate trial in which subjects exercised a considerably shorter time (69 min, 1.8-fold increase in GDF15). Interestingly, when subjects reached exhaustion, circulating GDF15 levels were ~1 ng/ml, considerably less than compared to ~2 ng/ml at exhaustion following high carbohydrate feeding. Together, these data underline that exercise duration is important for exercise-induced GDF15 levels. This raises the possibility that during low carbohydrate availability, comprising low muscle and liver glycogen stores, hindbrain sensitivity to GDF15 is increased in order to avoid behaviors that compromise glucose supply to the CNS. Such a central “set point” for the perceived exhaustion could involve an uncharacterized liver–hindbrain crosstalk with GDF15 as a key humoral signal.

A greater increase in circulating GDF15 after strenuous exercise (2 h or more) also could be related to muscle damage. However, the prolonged cycling was performed by highly trained athletes accustomed to this type of exercise. Further, we observed no correlation between changes in levels of plasma creatine kinase (CK), a marker of muscle damage, and changes in levels of GDF15 in response to a marathon run (Fig. 1f), ruling out this possibility. In a previous study, we measured GDF15 in arterial and venous blood sampled from the exercising leg and found similar GDF15 concentrations in both vessels, suggesting that GDF15 is not released from skeletal muscle during exercise in humans^10^. Therefore, other organs likely to secrete GDF15 during exercise. IL-6 is a well-established myokine with pleiotropic whole body effects that is rapidly released from skeletal muscle during exercise^17^. To probe if exercise-induced IL-6 secretion from muscle could trigger GDF15 release from other organs, we investigated plasma GDF15 in healthy humans infused with two doses of recombinant human IL-6 followed by a mixed meal test (Fig. S1a). Administration of IL-6 alone or in combination with a mixed meal did not impact circulating levels of GDF15. Another major anti-inflammatory cytokine induced by exercise is IL-10^18^. The induction of IL-10 in response to exercise is delayed compared to that of IL-6, and the magnitude of exercise-induced IL-10, like GDF15, is related to the duration of exercise^19^. Notably, we observed a strong correlation between the exercise-mediated increase in circulating IL-10 and exercise-induced GDF15 following a marathon run (Fig. 1g). However, in mice, treatment with IL-10 did not increase GDF15 plasma levels, and conversely, treatment with GDF15 did not increase IL-10, suggesting that there is no causal relationship between GDF15 and IL-10 (Fig. S1b–d). In summary, these data, from four independent human exercise studies, clearly underscore that different types of exercise increase circulating GDF15 levels and, with longer bouts of moderate- to high intensity exercise, GDF15 levels increase 4-5-fold.

### Effect of metabolic stressors on GDF15 in mice and humans

**Fig. 2:**
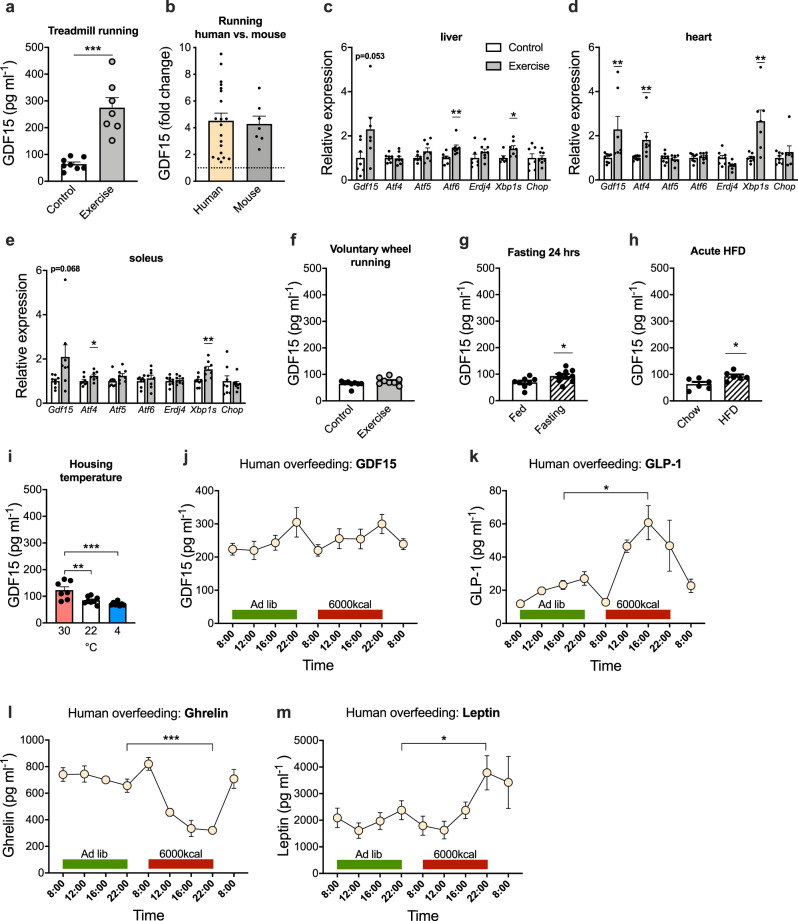
Effect of physiological metabolic stressors on circulating GDF15 in mice and humans. **a** Plasma GDF15 levels were measured in mice exposed to forced treadmill running until exhaustion (*n* = 7) compared to cage-matched sedentary controls (*n* = 8; *p* < 0.001). **b** Fold-change in circulating GDF15 after exhaustive endurance running in mice (based on Fig. 2a) vs. humans (based on Fig. 1c). **c–e** Relative mRNA levels of indicated genes in liver (*Aft6*, *p* = 0.0090; *Xbp1s*, *p* = 0.0301), heart (*Gdf15*, *p* = 0.0375; *Aft4*, *p* = 0.0229; *Xbp1s*, *p* = 0.0040), and skeletal muscle (soleus) (*Atf4*, *p* = 0.0402; *Xbp1s*, *p* = 0.0053) after forced treadmill running until exhaustion (*n* = 8) compared to cage-matched sedentary controls (*n* = 8; liver, *n* = 7). **f** Circulating GDF15 levels in mice following 3 weeks of voluntary running vs. non-running controls (*n* = 8) measured in plasma sampled 2 h into the dark phase. **g** Circulating GDF15 levels in mice after 24 h fasting (*n* = 9; *p* = 0.0384) compared to ad libitum fed mice (*n* = 8). **h** Circulating GDF15 levels in mice in response to 24 h of ad libitum, high-fat diet exposure compared to aged-matched control mice kept on chow diet (*n* = 6; *p* = 0.0202). **i** Circulating GDF15 levels in mice acclimatized to thermoneutrality (30 °C), kept at room temperature (22 °C), or mice exposed to chronic cold (4 °C) for 3 weeks (*n* = 8; 30 °C, *n* = 7; 30 °C vs. 22 °C, *p* = 0.0036; 30 °C vs. 4 °C, *p* < 0.001). **j**–**m** Plasma GDF15, GLP-1 (*p* = 0.0153), ghrelin (*p* < 0.001), and leptin (*p* = 0.0256) concentrations at indicated time points before, during, and after short-term overfeeding (6000 kcal in 14 h) in young healthy male human subjects (*n* = 5). Data are presented as mean ±  SEM, *P < 0.05; **P < 0.01; ***P < 0.001. **a**–**h** Two-tailed unpaired *t* test, except for *Gdf15* in **c**, where a Mann–Whitney test was applied. **i** Repeated measures One-way ANOVA. **j**–**m** Two-tailed paired *t* test between peak time-point for OF vs. corresponding ad lib time-point. Source data are provided as a Source Data file.

It was recently demonstrated that an increase in circulating GDF15, in response to metformin treatment, promotes weight loss in a GFRAL-dependent fashion^20,21^. Given that prolonged exercise leads to circulating GDF15 levels comparable to the GDF15 levels reported following metformin treatment, it raises the intriguing possibility that exercise-induced increases in endogenous GDF15 mediate the effects of intense exercise on transient food intake suppression^22,23^. To address this possibility mechanistically in mice, we established a forced running protocol on an inclined treadmill that resulted in a > 4-fold increase in plasma GDF15 levels post-exercise (Fig. 2a), similar to what we observed with longer duration, high intensity exercise in humans (Figs. 2b and  1c). Immediately after this treadmill exercise paradigm, *Gdf15* mRNA was increased 2-fold in liver, heart, and soleus muscle, suggesting that key tissues involved in substrate metabolism during exercise are contributing to the increased circulating GDF15 levels (Fig. 2c–e). The increased expression of *Gdf15* in these tissues coincided with an increase in markers related to the unfolded protein response (UPR) cellular stress pathway such as *Xbp1*, *Atf4*, and *Atf6* (Fig. 2c–e). Important to note, basal *Gdf15* expression is substantially higher in the liver compared to skeletal muscle and heart muscle (24-fold and 14-fold, respectively) (Fig. S2a). Thus, despite that an increase in *Gdf15* mRNA in human exercising muscle has been reported^24^, it remains unclear if GDF15 is a relevant myokine^10,25^. In contrast to forced treadmill running, voluntary wheel running does not increase circulating GDF15 levels, likely due its lower intensity and/or intermittent nature (Fig. 2f).

A high intensity exercise bout represents a transient physiological and metabolic stressor. To understand whether other forms of physiological and metabolic stressors trigger similar increases in circulating GDF15 levels, we assessed the effects of fasting, acute high-fat diet (HFD) feeding, and changes in ambient temperature on circulating GDF15 in mice and then evaluated the effect of acute, severe overfeeding (OF) in human subjects. Fasting for 24 h increased plasma GDF15 levels by 35% in mice (Fig. 2g). A change from chow diet to HFD resulted in 48% increase in plasma GDF15 levels 24 h following the diet switch (Fig. 2h). Mice acclimatized to thermoneutral conditions (30 °C) had 45% higher GDF15 levels compared to mice kept at standard housing temperature (22 °C), whereas chronic cold stress (4 °C), relative to 22 °C, had no effect on circulating GDF15 levels (Fig. 2i).

As a stress-responsive hormone, GDF15 might protect against severe OF via signaling food aversion^8^. To test this hypothesis, we measured plasma GDF15 levels in healthy humans before, during, and after they consumed 6000 kcal within 14 h. GDF15 levels showed a diurnal pattern with peak values around 10 pm, which is consistent with findings by Tsai et al^26^. However, severe short-term OF failed to elevate circulating GDF15 (Fig. 2j), arguing against a role of GDF15 in short-term physiological energy balance regulation. In contrast, canonical appetite hormones, GLP-1 (160% increase at 4 pm OF vs. 4 pm ad libitum), ghrelin (50% decrease at 10 pm OF vs. 10 pm ad libitum), and leptin (60% increase at 4 pm OF vs. 4 pm ad libitum) were markedly affected by OF (Fig. 2k–m). Overall, these canonical stressors of energy homeostasis only affected circulating GDF15 levels to a much lesser extent than exercise in mice and humans, suggesting that vigorous and prolonged exercise is especially effective at promoting acute GDF15 secretion.

### Divergent effects of pharmacological and physiological GDF15

**Fig. 3:**
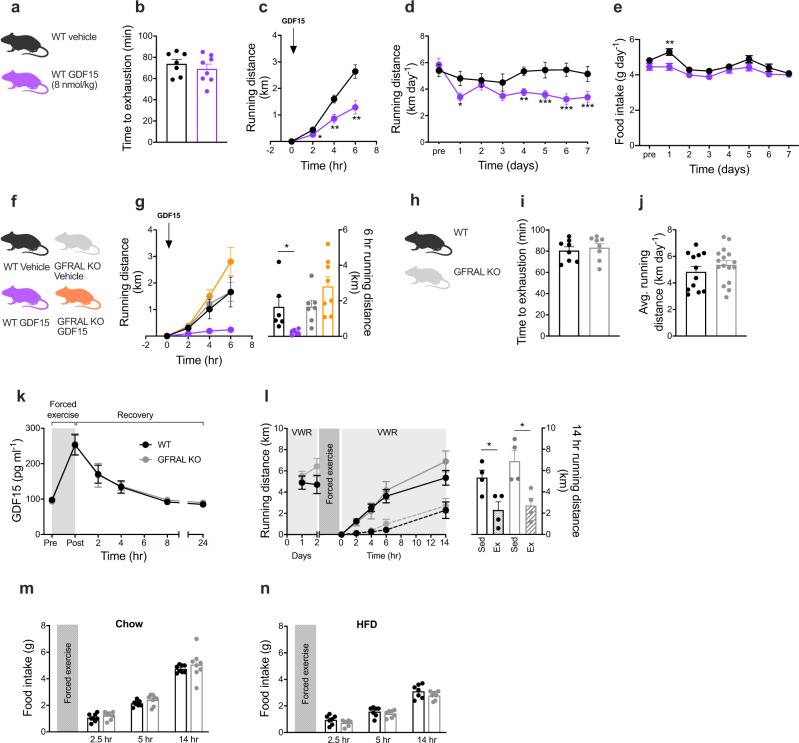
Effects of pharmacological GDF15 and exercise-induced GDF15 on food intake and running behavior. **a, b** Effect of single subcutaneous injection of rhGDF15 (8 nmol/kg bw, *n* = 8) or vehicle (*n* = 7) on forced treadmill running until exhaustion in mice. **c** Effect of single subcutaneous injection of rhGDF15 (8 nmol/kg bw, *n* = 16) or vehicle (*n* = 16) administered at the onset of the dark cycle on voluntary wheel running distance (2 h, *p* = 0.0210; 4 h, *p* = 0.0075; 6 h, *p* = 0.0036). **d, e** Effect of daily subcutaneous injections (at the onset of the dark cycle) of rhGDF15 (8 nmol/kg bw, *n* = 14) or vehicle (*n* = 14) on voluntary wheel running (day 1, *p* = 0.0140; day 4, *p* = 0.0042; day 5, *p* < 0.001; day 6, *p* < 0.001; day 7, *p* < 0.001) and food intake (day 1, *p* = 0.0032). **f**, **g** Effect of single subcutaneous injection of rhGDF15 (8 nmol/kg bw) or vehicle on voluntary wheel running in WT (vehicle *n* = 6; rhGDF15 *n* = 6, *p* = 0.0308) and GFRAL KO (vehicle *n* = 7; rhGDF15 *n* = 8) mice. **h, i** Forced treadmill running to exhaustion in WT (*n* = 8) and GFRAL KO (*n* = 8) mice. **j** Voluntary wheel running distance in WT (*n* = 12) and GFRAL KO (*n* = 16) mice **k** Plasma GDF15 values were measured after forced exercise to exhaustion on treadmill in WT (*n* = 13) and GFRAL KO (*n* = 11) mice. **l** Effect of forced exhaustive treadmill exercise on voluntary wheel running (VWR) in WT (*n* = 4 sedentary vs. *n* = 4 forced exercise, *p* = 0.0261) and GFRAL KO (*n* = 4 sedentary vs. *n* = 4 forced exercise, *p* = 0.0134) mice. **m** Effect of forced exhaustive treadmill exercise on chow in WT (*n* = 8) and GFRAL KO (*n* = 8) mice, and **n** HFD intake in WT (*n* = 7) and GFRAL KO (*n* = 7) mice. Data are presented as mean ±  SEM, *P < 0.05; **P < 0.01; ***P < 0.001. **b, g, i, j, l, m, n** Two-tailed unpaired *t* test. **c**, **d**, **e**, **k**, Repeated measures two-way ANOVA with Bonferroni multiple comparisons test. Source data are provided as a Source Data file.

To investigate if GDF15 modulates subsequent exercise performance or exercise behavior, we tested a single pharmacological dose of recombinant human GDF15 (rhGDF15) in mice (Fig. 3a) exposed to either controlled treadmill running or voluntary wheel running. Administration of rhGDF15, at a dose that suppressed acute feeding by ~50% in obese mice (Fig. S3a), had no influence on treadmill running to exhaustion in mice (Fig. 3b), suggesting that rhGDF15 has no adverse effects on the neuromuscular system or on cardiovascular fitness per se. In contrast, administration of rhGDF15 substantially lowered voluntary wheel running in mice adapted to running wheels (Fig. 3c). Decreases in voluntary running by rhGDF15 administration was sustained over a once daily treatment study for 7 days, by an average of -1.4 km/day (Fig. 3d). Notably, these effects on voluntary running were dissociated from the anorectic effects of rhGDF15 administration, which only induced a transient decrease in food intake with no effect on body weight (Figs. 3e and S3b). This implies that the effect of rhGDF15 on voluntary running behavior is more potent than the anorexigenic effects of rhGDF15. Our observation agrees with findings of a previous study showing that GDF15 administration more potently induces taste aversion compared to appetite suppression^8^. The exercise aversion following rhGDF15 treatment was absent in mice lacking the GDF15 receptor, GFRAL (Fig. 3f, g), underscoring that this behavior is mediated via the canonical GDF15-GFRAL hindbrain signaling pathway. Together these data emphasize that pharmacological GDF15 potently suppresses voluntary exercise, and that this is unrelated to an effect on treadmill exercise capacity. These findings extend the reported effects of pharmacological GDF15 on sickness-like behaviors, including taste aversion^8^, nausea, and emesis^9^.

To test the hypothesis that an endogenous increase in circulating GDF15 levels by controlled strenuous exercise is a physiological stressor that triggers aversive circuits via the brainstem to discontinue the activity, we exercised WT and GFRAL KO mice using forced treadmill running, after which mice were given free access to running wheels in their home cages. Of note, we observed similar body weights and similar fat and lean mass in WT and GFRAL KO mice on both chow and HFD (Fig. S3c–f). Further, prior to the treadmill running bout, we observed that exercise capacity and baseline voluntary wheel running activity were similar between WT and GFRAL KO mice with both groups running ~5 km/day (Fig. 3h–j). The induction and clearance of circulating GDF15 in response to treadmill running was likewise similar for WT and GFRAL KO mice (Fig. 3k), suggesting that endogenous GDF15 is not regulated through an endocrine feedback circuit involving GFRAL. Forced treadmill running substantially decreased voluntary running for several hours post exercise in both WT and GFRAL KO mice, indicating that this behavioral effect was independent of the endogenous GDF15-GFRAL signaling axis (Fig. 3l). Likewise, post exercise food intake was also similar between WT and GFRAL KO mice, indicating that forced exhaustive treadmill running failed to elicit GFRAL-dependent effects on appetite (Fig. 3m). Given that previous studies have found that GDF15 more potently regulates energy balance when the animals are on a HFD^21^, we assessed post exercise consumption of a palatable HFD in WT and GFRAL KO mice. However, similar to what we observed in chow-fed mice, we did not register any genotype differences in the immediate consumption of HFD following forced treadmill running (Fig. 3n).

## Discussion

The data presented here identify GDF15 as a physiological, exercise-induced, stress signal. Given the emerging understanding that the weight-lowering effects of pharmacological GDF15 cannot be dissociated from sickness-like behaviors^9^, we experimentally pursued the notion that endogenously-induced GDF15 in response to exercise stress contributes to exercise fatigue as a strategy to protect the organism and preserve energy. In line with this, we discovered that vigorous endurance exercise, lasting for more than 2 h, elicits circulating GDF15 levels comparable to those observed in patients suffering from mitochondrial disease^15^, infection^14^, and cancer^27^. Such pathological conditions are frequently accompanied by sickness-related anorexia and weight loss, and there is evidence linking GDF15 to non-homeostatic weight loss^4,9^. It is notable that the animal data presented here confirm that a pharmacological dose of GDF15 indeed inhibits feeding and reduces the motivation to exercise. In contrast, however, physiological induction of endogenous circulating GDF15 in response to prolonged strenuous exercise does not appear to be responsible for exercise-related aversion or acute suppression of food intake. Future studies are warranted to uncover this dissociation of the metabolic and behavioral consequences of pharmacological vs. physiological GDF15 and to define the role of GDF15 as an exerkine.

A limitation of this work relates to the translational relevancy of behaviors observed in rodents. Whereas we clearly demonstrate that pharmacological GDF15 reduces voluntary running in mice, it remains to be demonstrated that increased levels of GDF15 in humans lead to behavioral changes. Further, it is important to emphasize that the pharmacological doses of GDF15 used in most preclinical studies, including this one, increases GDF15 plasma levels beyond what are likely to be achieved by any physiological condition. Because other interventions conclusively demonstrate that a 2–5- fold increase in endogenous production of GDF15 is sufficient to elicit effects on energy balance^20,21^, we believe that increases in circulating GDF15 in response to exercise, mediate yet-to-be-determined physiological effects.

## Methods

### Human studies

To determine the impact of different types of exercise on circulating GDF15 levels in humans, we acquired existing plasma material from previous studies, and performed new studies (see Table S1 for study overview of human experiments).

#### Study 1: Endurance vs. resistance exercise

Ten healthy, young, moderately trained males participated in the study (age, 24 ± 3.2 years; BMI 23.7 ± 1.9 kg m^−2^; peak oxygen uptake (VO_2peak_) 55.7 ± 5.4 ml min^-1^ kg^-1^, [mean ± SD]). All subjects gave written consent to take part in the experimental procedures. Study methods have been described in detail previously^28^. Briefly, on two occasions separated by 1 week, subjects were instructed to cycle at 70% of VO_2peak_ for 1 h or follow a high-volume resistance exercise program for 1 h. Venous blood samples were drawn before exercise, immediately post-exercise, and at multiple time-points post-exercise. The study was approved by the local ethics committee of Copenhagen and Frederiksberg (H-17030450) and conformed to the standards described in the Helsinki Declaration.

#### Study 2: Exhaustive cycling in elite endurance athletes

Fifteen elite male triathletes (age, 27.0 ± 3.1 years; VO_2peak_ 66.1 ± 5.0 ml min^−1^ kg^−1^ [mean ± SD] were fully informed of any risks associated with the study before providing informed verbal and written consent. All experimental procedures have been described in detail elsewhere^29^. In short, participants received a standard diet before cycling at 73 ± 1% of their maximum heart rate for 4 h in the laboratory. During the first 4 h of recovery, subjects received either water or carbohydrate. Venous blood samples were drawn before exercise, after 2 h of exercise, and immediately post-exercise. Additional blood sampling was done 4 h and 24 h post-exercise. The study was approved by the ethics committee of the Region of Southern Denmark (Project ID: S-20090140) and conformed to the standards described in the Helsinki Declaration.

#### Study 3: Marathon run

Twenty healthy, moderately trained males were included in the study (age, 32.0 ± 8.5 years; BMI 24.7 ± 2.0 kg m^-2^; VO_2peak_ 52.5 ± 4.6 ml min^−1^ kg^−1^ [mean ± SD]). All subjects gave their written consent before entering the study. Venous blood samples were collected within the week before to the marathon and immediately after completion of the run. Additional sampling was done approximately 4 (2–5) and 9 (9-10) days after the marathon. All experimental procedures have been described in detail elsewhere^30^. The study was an observational study conducted in relation to Copenhagen Marathon 2018 and was approved by the Biomedical Ethical Committee of the Capital Region of Denmark (H-17041877) and conformed to the standards described in the Helsinki Declaration.

#### Study 4: Cycling to exhaustion on different dietary regimes

Eleven young, healthy males (age 25 ± 4 years; VO_2peak_ 59.4 ± 7.3 ml min^−1^ kg^−1^ [mean ± SD]) were included in the study. All subjects gave their written consent before entering the study. All experimental procedures have been described in detail elsewhere^31^. Briefly, on three occasions, each separated by 72 h, subjects were instructed to cycle at 75% of VO_2peak_ until exhaustion. For the 72 h leading up to the trials, subjects ingested either a standard diet, a low-carbohydrate diet (range: 0.2–0.3 g kg BM^−1^ day^−1^), or a high-carbohydrate diet (range: 7.4–9.7 g kg BM^−1^ day^−1^). All diets were isocaloric. Venous blood samples were taken pre- and post-exercise on each occasion. The study was approved by the Regional Committees on Health Research Ethics for Southern Denmark (S-20170198) and conformed to the standards described in the Helsinki Declaration.

#### Study 5: IL-6 infusion study

Seven young and healthy males (age 24 ± 1.1 years; BMI 24.1 ± 2.4 kg m^−2^ [mean ± SD]) participated in the study. All subjects gave their written consent before entering the study. Parts of this study have previously been published and more details can be found in Lang Lehrskov et al.^32^. On three different occasions, each separated by 7 days, subjects underwent 30 min infusion of either saline (placebo), low dose (1.5 μg) recombinant human (rh) IL-6, or high dose (15 μg) rhIL-6 through the antecubital vein. Immediately after infusion, a 2 h liquid mixed meal tolerance (15E% fat, 20E% protein, and 65E% carbohydrate) test (MMTT) was performed. Fasted venous blood samples were obtained before (0) and after (30 min) the infusion and at time points 60 and 120 min relative to the start of the MMTT. The study was approved by the ethical committee of the Capital Region of Denmark and conformed to the standards described in the Helsinki Declaration.

#### Study 6: Acute severe OF

Five healthy, lean males (age 33 ± 5.3 years; BMI 23.1 ± 1.1 kg m^−2^ [mean ± SD]) were recruited to the study. The purpose of the acute OF study, together with potential risks and﻿ inconveniences, was explained to all participants, and all gave signed consent prior to inclusion. Subjects were followed on three consecutive days. Subjects consumed their habitual diet on day 1 and, on day 2, were exposed to a 6000 kcal hypercaloric diet (37E% fat, 12E% protein, and 51E% carbohydrate). Venous blood was sampled at 8:00 a.m., 12:00 a.m., 4:00 p.m., and 10:00 p.m. at day 1 and day 2 and at 8.00 a.m. on day 3 after the hypercaloric diet. The study protocol was submitted to the Ethical Committee of the Capital Region of Denmark for assessment. The Ethical Committee of the Capital Region of Denmark concluded that the study protocol proposal was not a health research project as defined by the committee law, §2, and therefore the study did not require formal approval and thus gave permission to execute the study (Journal ID: 18043249). The study conformed to the standards described in the Helsinki Declaration.

### Animal studies

#### Animals

Wild-type (WT) C57BL/6 J male and female mice were obtained from (Janvier, FR). The GFRAL knockout (KO) and WT littermates were generated as previously reported^33^. One week before experimental treatment, mice were acclimatized to single housing. All experiments were done at 22 °C (35–55% humidity) with a 12:12‐h light–dark cycle. Mice had free access to water and chow diet (Altromin 1324, Brogaarden, DK), or when indicated a HFD (D12331; Research Diets). All experiments were carried out in accordance with regulations regarding the care and use of experimental animals, and the experimental procedures were approved by the Danish Animal Experimentation Inspectorate (2018-15-0201-01457).

#### Forced treadmill running

One week before the experimental day, 12–14 weeks old male mice were familiarized to treadmill (TSE Systems, D) three times (10 min at 10.2 m min^−1^), and subsequently exposed to a running paradigm of 10 min at 6 m min^−1^ then 40 min at 17.4 m min^−1^ (50% of max speed) with a slope at 10° followed by gradually increased speed (0.6 m min^−1^) until exhaustion. Exhaustion was defined when mice fell back to the grid three times within 30 s. Mice were sacrificed immediately after exercise. Blood was immediately centrifuged at 5000×*g* at 4 °C for 10 min and stored at −80 °C for later analyses. Tissues were rapidly frozen on dry ice and stored at −80 °C for later analyses. Another cohort of 8 weeks old male WT and GFRAL KO mice, completed the same exercise protocol, and blood samples were drawn from the tail before treadmill exercise, immediately after and at 2, 4, 8, and 24 h post exercise for measuring plasma GDF15 levels.

#### Voluntary running

Eight–ten weeks old male mice were single-housed in cages equipped with running wheels (23 cm in diameter, Techniplast, I). The amount of bedding was reduced in order to avoid wheel blockade. Running distance was measured by a computer (Sigma Pure 1 Topline 2016, D), and after 1 week of familiarization to the wheels, running distance was measured daily for 3 weeks. After 3 weeks, blood samples from the tail were taken 2 h after onset of the dark cycle.

#### Fasting and HFD-feeding

Eight weeks old male mice were single-housed and fed a regular chow diet. For the fasting study, mice were randomized to either fasting (24 h) or remaining on chow diet. For the HFD-switch study, chow fed mice were randomized to either staying on chow diet (24 h) or having free access to a HFD for 24 h. Blood samples from the tail were taken for measuring GDF15 concentration. Ten weeks old male WT and GFRAL DIO mice had free access to a HFD (Research Diets d12331) for 6 months, following which body composition was measured by magnetic resonance imaging (EchoMRI-4in1Tm, Echo Medical system LLC, USA).

#### Housing Temperature

Male mice fed on chow diet were randomized to housing at 30 °C (32 weeks of age), at 4 °C (23 weeks of age), or remained at 22 °C (23 weeks of age). After 3 weeks, trunk blood was collected for measuring GDF15 concentration.

#### In vivo pharmacology and exercise motivation

Pharmacokinetic properties of recombinant human (rh) GDF15 (Novo Nordisk) in mice were evaluated by subcutaneous administering of 8 nmol kg^−1^ (0.2 mg kg^−1^) or 0.8 nmol kg^−1^ (0.02 mg kg^−1^), respectively (Fig. S1b**)**. Plasma was collected at baseline and 2, 4, 8, and 24 h following treatment. For the exercise treadmill exhaustion test, 12–14 weeks old female WT mice were administered 8 nmol kg^−1^ (0.2 mg kg^−1^) at 5 µl per gram body weight or vehicle solution 2 h before treadmill running (identical to above described protocol). For the effect of rhGDF15 on voluntary wheel running, after 1 week of familiarization to running wheels, 8–10 weeks old female WT mice were randomized to receive either subcutaneous injection of rhGDF15 (8 nmol kg^−1^) or vehicle once daily for seven consecutive days. Injections was done each day within the last 60 min of the light cycle. After the first dosing, running distance was monitored at 2, 4, and 6 h, and, for the remaining part of the study, distance, body weight, and food intake were measured once daily. For assessment of GFRAL-dependent effects of rhGDF15 on voluntary wheel running, 12–14 weeks old male WT and GFRAL KO mice were subcutaneously administered rhGDF15 at 8 nmol kg^−1^ (5 µl per gram body weight) or vehicle control prior to gaining access to running wheels. Distance was measured 2, 4, and 6 h following the pharmacological intervention.

Exercise time to exhaustion in WT vs. GFRAL KO mice was performed according to the protocol described above. Average running distance in WT and GFRAL KO was measured in 10–16 weeks old male mice with free access to running wheels over 2 weeks.

Pharmacokinetic properties of recombinant mouse IL-10 (Novo Nordisk) were evaluated by subcutaneously administering 40 μg kg^−1^ or 400 μg kg^−1^ (Fig. S1b**)**. Plasma was collected at baseline and 2, 4, 8, and 24 h following treatment.

#### Forced exercise: Subsequent effects on voluntary exercise and food intake

Eight–fourteen weeks old male WT and GFRAL KO mice were exercised to exhaustion on a treadmill (as described previously) immediately before the beginning of the dark cycle after which they were returned to their cages with running wheel, for which they had previously been adapted. Running distance was monitored at 2, 4, 6, and 14 h post exercise.

In a separate experiment, the same forced exercise protocol was applied, and animals were returned to their home cages with no running wheels. Assessment of food intake was done at 2.5, 5, and 14 h post exercise. This exercise-feeding study was repeated in animals that were exposed to a HFD (Research Diets d12331) for 5 days prior to study start.

### Biochemistry

GDF15 was measured in human plasma or serum using the Quantikine ELISA Human GDF-15 Immunoassay (ELISA, R&D systems, catalog no. DGD150). IL-10 was determined in single determinations using multi-spot immunoassay (V-PLEX, Meso Scale Discovery, Rockville, MD, USA) on EDTA treated plasma. CK was determined in lithium–heparin treated plasma using Cobas. Leptin was determined using a leptin ELISA assay (ELISA, R&D systems, catalog no. DLP00). GLP-1 was determined using a V-PLEX assay (Meso Scale Discovery, GLP-1 total kit, catalog no. L4503PA-1). Ghrelin was determined using total ghrelin ELISA assay (Merck Millipore, catalog no. EZGRT-89K). In mice, plasma samples were analyzed using Quantikine ELISA Mouse GDF15 Immunoassay (ELISA, R&D systems, catalog no. MGD150) and Mouse IL-10 immunoassay (ELISA, R&D systems, catalog no. M1000). The ELISA assays were used according to the protocol provided by the manufacturer.

### RNA extraction & cDNA synthesis

Tissue was quickly dissected and frozen on either dry ice or liquid nitrogen and stored at −80 °C. Tissue was homogenized in a Trizol reagent (QIAzol Lysis Reagent, Qiagen) using a stainless steel bead (Qiagen) and a TissueLyser LT (Qiagen) for 3 min at 20 Hz. Then, 200 μl chloroform (Sigma-Aldrich) was added and tubes were shaken vigorously for 15 sec and left at RT for 2 min, followed by centrifugation at 4 °C for 15 min at 12,000×*g*. The aqueous phase was mixed 1:1 with 70% ethanol and further processed using RNeasy Lipid Mini Kit following the instructions provided by the manufacturer. For muscle tissue, the lysis procedure, described the enclosed protocol in the Fibrous Tissue Mini Kit (Qiagen), was followed. After RNA extraction, RNA content was measured using a NanoDrop 2000 (Thermo Fisher) and 500 ng of RNA was converted into cDNA by mixing FS buffer and DTT (Thermo Fisher) with Random Primers (Sigma-Aldrich) and incubated for 3 min at 70 °C followed by addition of dNTPs, RNase out, Superscript III (Thermo Fisher) and placed in a thermal cycler for 5 min at 25 °C, 60 min at 50 °C, 15 min at 70 °C, and kept at −20 °C until further processing.

### qPCR

SYBR green qPCR was performed using Precision plus qPCR Mastermix containing SYBR green (Primer Design, #PrecisionPLUS). For primer sequences, see Table S2. qPCR was performed in 384-well plates on a Light Cycler 480 Real-Time PCR machine using 2 min preincubation at 95 °C followed by 45 cycles of 60 sec at 60 °C and melting curves were performed by stepwise increasing the temperature from 60 to 95 °C. Quantification of mRNA expression was performed according to the delta-delta Ct method.

### Statistical analyses

Statistical analyses were performed in Graphpad Prism version 8 and 9. For comparing multiple groups, one- or two-way ANOVA with Bonferroni post hoc multiple comparisons test was used. When comparing two groups, a Students *t* test was used. When appropriate, ROUT method was used to detect outliers. Unless otherwise stated, all data are presented as mean ± SEM. *P* < 0.05 was considered statistically significant.

### Reporting Summary

Further information on research design is available in the Nature Research Reporting Summary linked to this article.

## Supplementary information

Supplementary Information

Reporting Summary

## Data Availability

All data that support the findings of this study are available from the corresponding author upon reasonable request. Source data are provided with this paper.

## Source data

Source Data
