# Supplementary material for: Pharmacological but not physiological GDF15 suppresses feeding and the motivation to exercise
Source: Nat Commun. 2021 Feb 15;12:1041. doi: 10.1038/s41467-021-21309-x (PMC7884842; doi:10.1038/s41467-021-21309-x)
Supplement: Supplementary file 1 — Supplementary Information [file 41467_2021_21309_MOESM1_ESM.pdf]

SUPPLEMENTARY INFORMATION

Pharmacological but not physiological GDF15 suppresses feeding and the motivation to exercise

Anders B. Klein *et al*

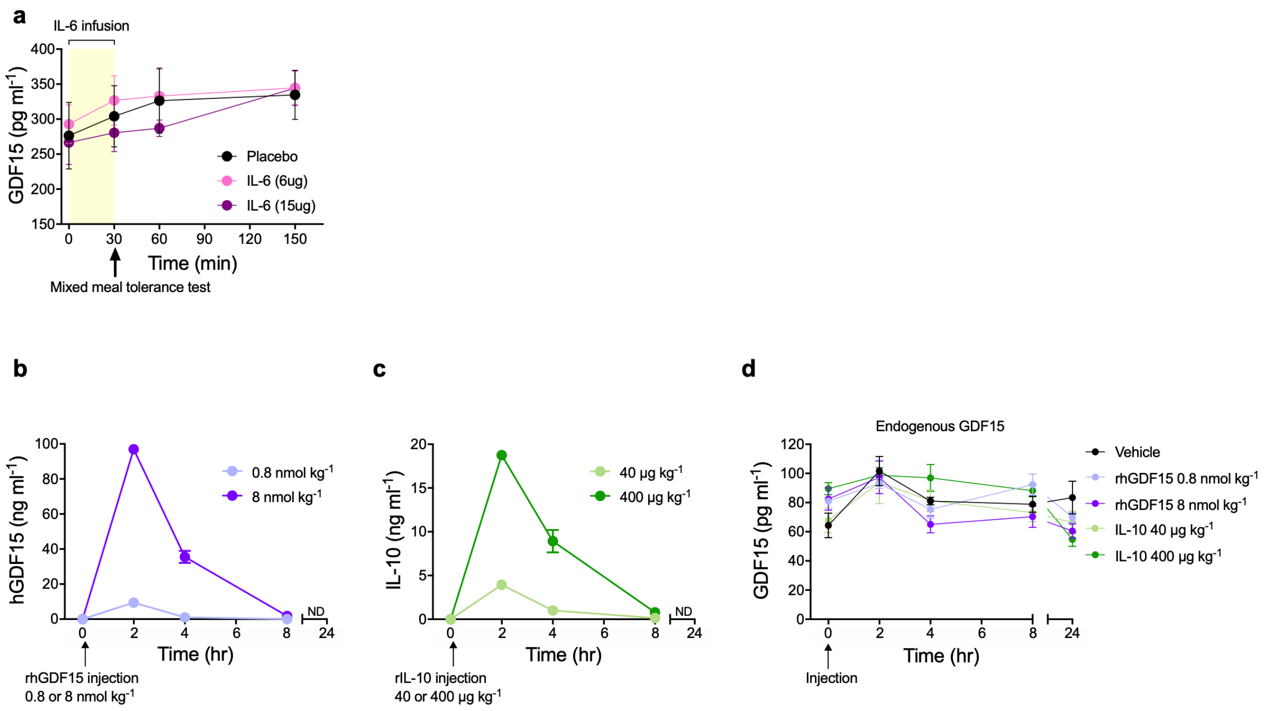

Supplementary Figure 1 | Effect of rhIL-6 infusion on circulating GDF15 levels in healthy humans and effects of rhGDF15 and rmIL-10 administrations on circulating GDF15 in mice. **a**, Plasma GDF15 concentration during IL-6 infusion followed by a mixed meal tolerance test, n=7. **b-d**, Plasma GDF15 levels after a single subcutaneous injection of either vehicle (n=4), rhGDF15 (0.8 nmol kg^{-1}, n=4 or 8 nmol kg^{-1}, n=4) or IL-10 (40 µg kg^{-1}, n=4 or 400 µg kg^{-1}, n=4). Data are presented as means ± SEM. Source data are provided as a Source Data file.

a

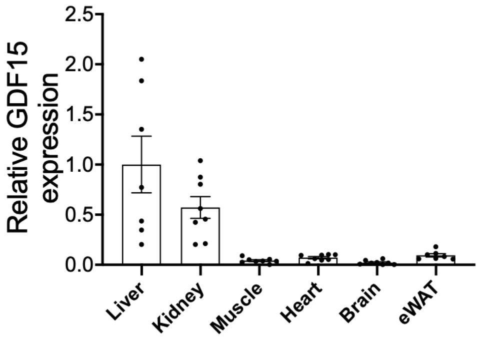

Supplementary Figure 2 | *Gdf15* mRNA expression in mouse tissues. a, Mouse tissue panel of *Gdf15* expression. *Gdf15* mRNA was normalized to reference gene *Rpl13a* and subsequently the normalized tissue *Gdf15* enrichment was compared relative to liver expression. n=8 (liver, n=7 and eWAT, n=7). Data are presented as means ± SEM. Source data are provided as a Source Data file.

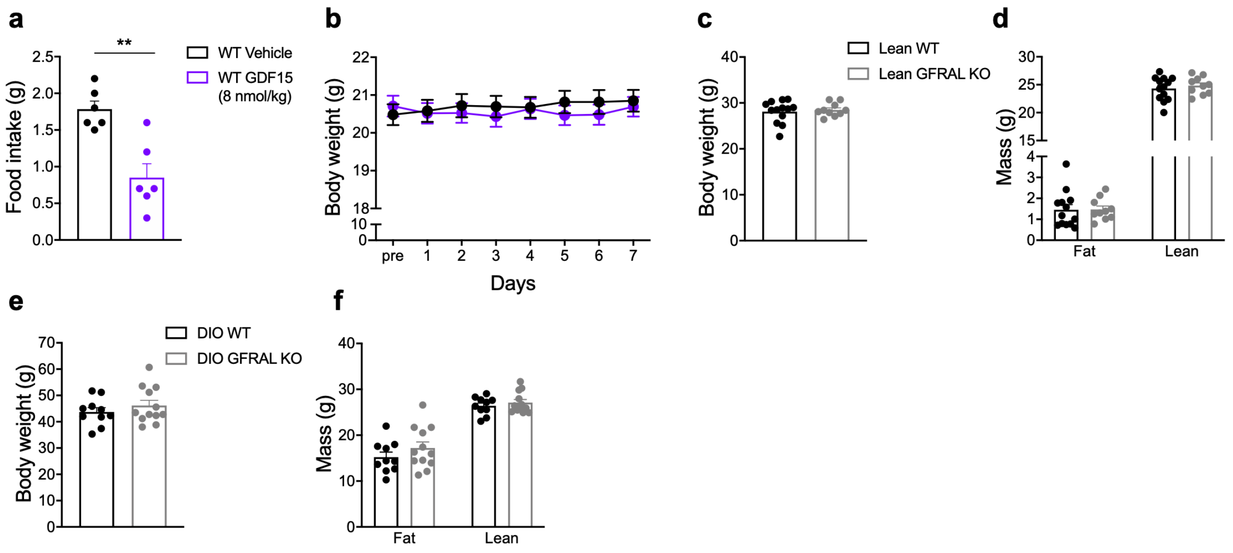

SupplementaryFigure 3 | Effect of rhGDF15 administration on overnight feeding in DIO mice, on body weight during a 7-day voluntary running study in chow-fed mice, and on body composition in WT and GFRAL KO mice on chow and HFD respectively. a, Overnight food intake of diet-induced obese (DIO) mice acutely treated with vehicle (n=6) or rhGDF15 (n=6, p=0.0018). **b**, Body weight in lean mice treated daily with vehicle (n=16) or rhGDF15 (n=15) while having free access to running wheels. **c**, Body weight and **d**, Body composition in lean WT (n=13) and GFRAL KO (n=10) mice. **e**, Body weight and **f**, body composition in DIO WT (n=10) and GFRAL KO (n=12) mice. Data are presented as means ± SEM. ** P < 0.01, two-tailed unpaired t-test. Source data are provided as a Source Data file.

Supplementary Table 1. Plasma samples from studies on human subjects

Human Study	Figure	Intervention	Reference
Study 1	Fig.1a	Endurance vs. resistance exercise	25
Study 2	Fig.1b	Cycling	26
Study 3	Fig.1c	Marathon run	27
Study 4	Fig.1d	Cycling and diet interventions	29
Study 5	Fig.S1	IL-6 infusion	28
Study 6	Fig.2J-M	Acute overfeeding	This study

Supplementary Table 2. Primers for RT-qPCR

**Primers for qPCR**	**Sequence**
GDF15 F	CCGAGAGGACTCGAACTCAG
GDF15 R	ACCCCAATCTCACCTCTGGA
ATF4 F	GATGAGCTTCCTGAACAGCG
ATF4 R	GCCAAGCCATCATCCATAGC
ATF5 F	CTACCCCTCCATTCCACTTTC
ATF5 R	TTCTTGACTGGCTTCTCACTTGTG
ATF6 F	TTATCAGCATACAGCCTGCG
ATF6 R	CTTGGGACTTTGAGCCTCTG
Xbp1s F	CTGAGTCCGAATCAGGTGCAG
Xbp1s R	GTCCATGGGAAGATGTTCTGG
Erdj4 F	ATAAAAGCCCTGATGCTGAAGC
Erdj4 R	GCCATTGGTAAAAGCACTGTGT
CHOP F	CCACCACACCTGAAAGCAGAA
CHOP R	AGGTGAAAGGCAGGGACTCA
Rpl13a F	GGAGGGGCAGGTTCTGGTAT
Rpl13a R	TGTTGATGCCTTCACAGCGT
