# Supplementary material for: Pharmacological but not physiological GDF15 suppresses feeding and the motivation to exercise
Source: Nat Commun. 2021 Feb 15;12:1041. doi: 10.1038/s41467-021-21309-x (PMC7884842; doi:10.1038/s41467-021-21309-x)
Supplement: Supplementary file 2 — Reporting Summary [file 41467_2021_21309_MOESM2_ESM.pdf]

nature research

Corresponding author(s): Christoffer Clemmensen

Last updated by author(s): 2020-Dec-17

Reporting Summary

Nature Research wishes to improve the reproducibility of the work that we publish. This form provides structure for consistency and transparency in reporting. For further information on Nature Research policies, see our Editorial Policies and the Editorial Policy Checklist.

Statistics

For all statistical analyses, confirm that the following items are present in the figure legend, table legend, main text, or Methods section.

n/a	Confirmed	
☐	☒	The exact sample size (n) for each experimental group/condition, given as a discrete number and unit of measurement
☐	☒	A statement on whether measurements were taken from distinct samples or whether the same sample was measured repeatedly
☐	☒	The statistical test(s) used AND whether they are one- or two-sided<br>*Only common tests should be described solely by name; describe more complex techniques in the Methods section.*
☐	☒	A description of all covariates tested
☐	☒	A description of any assumptions or corrections, such as tests of normality and adjustment for multiple comparisons
☐	☒	A full description of the statistical parameters including central tendency (e.g. means) or other basic estimates (e.g. regression coefficient) AND variation (e.g. standard deviation) or associated estimates of uncertainty (e.g. confidence intervals)
☐	☒	For null hypothesis testing, the test statistic (e.g. F, t, r) with confidence intervals, effect sizes, degrees of freedom and P value noted<br>*Give P values as exact values whenever suitable.*
☒	☐	For Bayesian analysis, information on the choice of priors and Markov chain Monte Carlo settings
☒	☐	For hierarchical and complex designs, identification of the appropriate level for tests and full reporting of outcomes
☐	☒	Estimates of effect sizes (e.g. Cohen's d, Pearson's r), indicating how they were calculated

Our web collection on statistics for biologists contains articles on many of the points above.

Software and code

Policy information about availability of computer code

Data collection	No custom made code or algorithm was used in the manuscript.
Data analysis	No custom made code or algorithm were used in the manuscript. Statistical analysis were performed using GraphPad Prism (versions 8 and 9)

For manuscripts utilizing custom algorithms or software that are central to the research but not yet described in published literature, software must be made available to editors and reviewers. We strongly encourage code deposition in a community repository (e.g. GitHub). See the Nature Research guidelines for submitting code & software for further information.

Data

Policy information about availability of data

All manuscripts must include a data availability statement. This statement should provide the following information, where applicable:

- Accession codes, unique identifiers, or web links for publicly available datasets
- A list of figures that have associated raw data
- A description of any restrictions on data availability

All data that support the findings of this study are available from the corresponding author upon reasonable request.

Field-specific reporting

Life sciences study design

All studies must disclose on these points even when the disclosure is negative.

Sample size	Sample sizes for exercise experiments in mice were based on previous experience with this type of studies (e.g. Nicolaisen et al., 2020, FASEB). The number of animals used is chosen based on the variability observed using these paradigms. Sample sizes in the human studies, were led by the primary design described in the primary publication (Study 1: Morville et al., 2019, JCI Insight; Study 2: Gejl et al., 2014, Med Sci Sports Exer; Study 3: Larsen et al., 2020, J Sports Sci; Study 4: Jensen et al., 2020, J. Physiology; Study 5: Lang Lehrskov et al., 2018, Cell Metabolism). Human Study 6 has not been published yet, but sample size is based on previous measurements of GDF15 (Kleinert et al., 2018, Molecular Metabolism) and other hormones involved in metabolism (Morville et al., 2019; JCI Insight).
Data exclusions	To identify outliers, ROUT method (GraphPad Prism) was applied. On rare occasions, a blood or tissue sample was missing due to technical challenges in the sampling procedure. All values are presented in the Source Data File. Here follows a complete overview of outliers and missing values: Figure 1c, 3 missing values; Figure 1d, 1 missing value, 1 outlier; Figure 1e, 1 outlier; Figure 1f, 1 outlier; Figure 1g, 2 outliers. Figure 2a, 1 missing value, Figure 2c, 3 missing values, 1 outlier; Figure 2i, 1 missing value; Figure 3d, 1 missing value; Figure 3k, 2 missing values; Figure S1b, 1 mouse excluded (injection failed), 2 missing values; Figure S1c, 1 mouse excluded (injection failed); Figure S2, 1 outlier, 1 missing value.
Replication	All data presented are reproducible. Key mouse experiments were performed at least twice. The human studies were not feasible to repeat due to their extensive nature. However, we are convinced that these data are reproducible. As example, Figure 1a, 1b, 1c, and 1d report - by different means - the exact same biological phenomenon, that exercise increases GDF15 in plasma of moderately trained and highly trained individuals.
Randomization	Animals were randomly distributed into experimental groups based on either body weight or voluntary running distance.
Blinding	All measurements of plasma GDF15 were performed blinded. Human studies were not possible to blind due to the close interaction with the subjects involved (e.g. blood sampling following a marathon run). Mouse experiments were blinded whenever possible, but tissue dissection, blood sampling and measurements of performance from sedentary and exercise mice was not feasible to blind. Samples used for qPCR measurements were blinded to the experimenter.

Reporting for specific materials, systems and methods

We require information from authors about some types of materials, experimental systems and methods used in many studies. Here, indicate whether each material, system or method listed is relevant to your study. If you are not sure if a list item applies to your research, read the appropriate section before selecting a response.

Materials & experimental systems

n/a	Involved in the study	
☒	☐	Antibodies
☒	☐	Eukaryotic cell lines
☒	☐	Palaeontology and archaeology
☐	☒	Animals and other organisms
☐	☒	Human research participants
☒	☐	Clinical data
☒	☐	Dual use research of concern

Methods

n/a	Involved in the study	
☒	☐	ChIP-seq
☒	☐	Flow cytometry
☒	☐	MRI-based neuroimaging

Animals and other organisms

Policy information about studies involving animals; ARRIVE guidelines recommended for reporting animal research

Laboratory animals	Wild-type (WT) C57BL/6J male and female mice were obtained from (Janvier, FR). The GFRAL knockout (KO) and WT littermates were generated as previously reported (Frikke-Schmidt et al. Mol Metab, 2019). Age of mice used ranged from 8 to 32 weeks, specified for each experiment in the Methods section. Mice were housed at ambient humidity ranging from 35-55%; with ad-libitum chow or high-fat diet in 12:12 hour light/dark cycle.
Wild animals	The study did not involve wild animals
Field-collected samples	The study did not involve collected samples from the field.
Ethics oversight	All experiments were approved by the Danish Animal Experimentation Inspectorate.

Note that full information on the approval of the study protocol must also be provided in the manuscript.

Human research participants

Policy information about studies involving human research participants

Population characteristics	Study 1: 24 ± 3.2 years, moderately trained, healthy males (n=10)<br>Study 2: 27 ± 3.1 years, elite triathletes, healthy males (n=15)<br>Study 3: 32 ± 8.5 years, moderately trained, healthy males (n=20)<br>Study 4: 25 ± 4 years, healthy males (n=11)<br>Study 5: 24 ± 1.1 years, healthy males (n=7)<br>Study 6: 33 ± 5.3 years, healthy males (n=5)
Recruitment	Participants were recruited by local advertisement. For Human Study 2, elite athletes were recruited. For Human Study 3, people were training for the Copenhagen Marathon. In Human Study 4, 5 and 6, we are aware of the risk of selection bias towards healthy young well-trained males, who is more likely to volunteer for this type of experiments. Importantly, this potential bias is unlikely to influence the results of Human Studies 4, 5 and 6.
Ethics oversight	Human study 1 was approved by the local ethics committee of Copenhagen and Frederiksberg (H-17030450). Human study 2 was approved by the ethics committee of the Region of Southern Denmark (Project ID: S-20090140). Human study 3 was approved by the Biomedical Ethical Committee of the Capital Region of Denmark (H-17041877). Human study 4 was approved by The Regional Committees on Health Research Ethics for Southern Denmark (S-20170198). Human study 5 was approved by the ethical committee of the Capital Region of Denmark. The Ethical Committee of the Capital Region of Denmark concluded that study 6 did not required formal approval and thus gave permission to execute the study (Journal ID: 18043249). All studies conformed to the standards described in the Helsinki Declaration.

Note that full information on the approval of the study protocol must also be provided in the manuscript.
